# Depletion of Nuclear Poly(A) Binding Protein PABPN1 Produces a Compensatory Response by Cytoplasmic PABP4 and PABP5 in Cultured Human Cells

**DOI:** 10.1371/journal.pone.0053036

**Published:** 2012-12-31

**Authors:** Rumpa Biswas Bhattacharjee, Jnanankur Bag

**Affiliations:** Department of Molecular and Cellular Biology, University of Guelph, Guelph, Ontario, Canada; Korea University, Republic of Korea

## Abstract

**Background:**

In vertebrates, poly(A) binding protein (PABP) is known to exist in five different isoforms. PABPs are primarily cytosolic with the exception of the nuclear PABP (PABPN1), which is located in the nucleus. Within the nucleus, PABPN1 is believed to bind to the poly(A) tail of nascent mRNA and along with cleavage and polyadenylation specificity factor (CPSF) define the length of the newly synthesized poly(A) tail.

**Methodology/Principal Findings:**

The cellular role of PABP1 has been extensively studied over the years; however, the function of other PABPs remains poorly defined. In order to understand the role of PABPN1 in cellular mRNA metabolism and it’s interrelation with other PABPs, we depleted PABPN1 using RNAi in HeLa and HEK293 cells. Our results show that PABPN1 depletion did not have any effect on the poly(A) tail length, nuclear export of mRNA, mRNA translation, and transcription. Rather, PABPN1 depletion resulted in a compensatory response as observed by increased level of PABP5 and nuclear accumulation of PABP4. In addition, PABP4 was associated with the poly(A) tract of pre-mRNA and CPSF in PABPN1 depleted cells. Nevertheless, PABPN1 depletion significantly affected cell survival as evidenced by an increase in apoptosis markers: phosphorylated p53 and PUMA and as judged by the expression of ER stress marker GRP78.

**Conclusion:**

Our results suggest that although function of PABPN1 may be compensated by nuclear translocation of PABP4 and perhaps by increase in the cytoplasmic abundance of PABP5, these were not sufficient to prevent apoptosis of cells. Thus PABPN1 may have a novel anti apoptotic role in mammalian cells.

## Introduction

Mammalian nuclear poly(A) binding protein (PABPN1) is a highly conserved nuclear RNA binding protein with specificity towards the poly(A) tract of eukaryotic mRNAs. It consists of one typical RNA recognition motifs (RRM) domain with consensus RNP1 and RNP2 motifs in the central region of the polypeptide, and an arginine rich C- terminal domain [1]. Both RNP domains and the C-terminal region of PABPN1 are required for binding to RNA and its polypeptide partners respectively. Interestingly, the amino acid sequence of the RNP domain of PABPN1 has no homology with the RNA binding domain of the cytoplasmic poly(A)-binding protein PABPC1 or other RNA binding proteins [1]. However, recent crystal structure analyses of human PABPN1 suggest that PABPN1 RRM still adopts a fold similar to canonical RRM structure consisting of a four stranded antiparallel β-sheet structure spatially arranged in the order of β4β1β3β2. The fold of the third loop and dimerization of the crystal are distinct features of PABPN1 [2].The nuclear localization signal is located between amino acids 289–306 and overlaps with the oligomerization domain [3], [4]. Mammalian PABPN1 contains an alanine tract consisting of twelve alanines after the first methionine at the N-terminal end which makes it prone to aggregate formation. This polyalanine tract, however, is not conserved, and is absent in Drosophila PABPN1 without any detectable loss of cellular function [5].

Results of biochemical studies suggest that the main cellular function of PABPN1 is to stimulate the elongation of poly(A) tract of eukaryotic mRNA, and control its length [6]. After the addition of first ten adenine residues by poly(A) polymerase, PABPN1 binds to it as a monomer. Additional PABPN1 assembles on the poly(A) tract at a packing density of 15 adenines per PABPN1 molecule [6], [7], [8] as the length of the tract progressively increases. Both PABPN1 and cleavage and poly adenylation specific factor (CPSF) stimulate the activity of poly(A) polymerase by mutually stabilizing their interaction with mRNA. CPSF and PABPN1 can stimulate the polyadenylation by poly(A) polymerase independently, but the extension of the 3′ end is much faster when both are present. When the poly(A) tail length reaches 250–300 nucleotides, further extension of the poly(A) tract becomes very slow [6]. The oligomerization of PABPN1 is functionally important and may serve as a molecular ruler to determine the length of the poly(A) tract [9]. The wild type PABPN1 exists in equilibrium as monomers, dimers and oligomers and filamentous complexes [10]. Poly alanine expansion mutations have been found in patients with Oculopharyngeal muscular dystrophy (OPMD). The OPMD mutant PABPN1 shows enhanced aggregation and forms nuclear inclusions in the muscle of affected individuals. However, no loss of cellular function due to this mutation has been detected [11]. PABPN1 associates with RNA polymerase II along the chromatin axis before or shortly after the transcription initiation, and the assembly of PABPN1 on the poly(A) tract may be coupled to transcription [12]. PABPN1 remains associated with the released mRNA-protein complex (mRNP) until it reaches the cytoplasmic side of the nuclear pore. Since very little PABPN1 is present in the cytoplasmic side of the nuclear envelope, it has been proposed that during or shortly after passage through the nuclear pore PABPN1 is displaced by PABP1 [3], [4], [13], [14]. PABPN1 can also interact with the SKI-binding polypeptide (SKIP) transcription factor and stimulate myogenesis [15]. Depletion of PABPN1 in myoblasts prevents myogenesis and reduces the length of the poly(A) tract of mRNAs [16]. In addition, PABPN1 depletion also affects cell proliferation in myoblasts and fibroblasts [16].

In contrast to the presence of only one nuclear poly(A) binding protein, vertebrates express several cytoplasmic PABPs with conserved RNA binding motifs [17]. Amongst the cytoplasmic PABP family the PABP1 (also known as PABPC1) has been most intensively studied. Four distinct RNA RRMs are located at the N-terminal region of PABP1 while the C-terminal region contains both a variable proline rich oligomerization domain and a conserved PABC domain with five α helices involved in binding to other polypeptides [17]. The other members of cytoplasmic PABP family are t-PABP also known as PABP2 in mouse and PABP3 in humans, PABP4 (or inducible PABP) and PABP5 which are separate gene products but are highly homologous to PABP1. All four cytoplasmic PABPs have four RRMs at their N-terminal ends and with the exception of PABP5, have a proline rich linker and a PABC domain [17]. PABP5 is a smaller polypeptide than all other cytoplasmic PABPs and is missing the entire C-terminal end. Very little information regarding the biochemical functions of these cytoplasmic PABPs are known. Antisense morpholino oligodeoxynucleotide mediated knock down experiments in *Xenopus laevis* suggests that both PABP1 and PABP4 are required for their normal development [18]. In addition, rescue studies [18] showed that PABP4 could not restore the effect of PABP1 knock down on development. Therefore, it is likely that each PABP has distinct functions in vertebrates.

Deletion of yeast pabp2 gene, which is the homolog of mammalian PABPN1, leads to extension of poly(A) tract of mRNAs [19], and accumulation of polyadenylated small nucleolar RNA (snoRNA). It has been recently shown that yeast nuclear PABP promotes snoRNA synthesis by recruiting exosomes for trimming its poly(A) tail [20]. Silencing of PABPN1 expression in mouse muscle cells in culture with RNAi resulted in shortening of poly(A) tail of mRNA and impaired export of processed transcripts to the cytoplasm [16]. In addition, another study reported that in absence of PABPN1 selection of proximal alternative cleavage and polyadenylation site is favored in human cells [21]. Here we report, depletion of PABPN1 using RNAi in HeLa cells did not lead to shortening of the poly (A) tract, and showed no detectable effect on the mRNA accumulation in the cytoplasm, mRNA translation, and transcription. PABPN1 depletion resulted in a compensatory response by preferential increase in the abundance of PABP5 and nuclear accumulation of PABP4. Furthermore, PABP4 was associated with the poly(A) tract of mRNA and CPSF in PABPN1 depleted cells. However, cell survival was significantly affected by increased apoptosis of PABPN1 depleted cells. Increased level of phosphorylated p53 and PUMA was seen in PABP depleted cells and as judged by the induction of GRP78 expression these cells were undergoing ER stress. Our results suggest that function of PABPN1 may be compensated by nuclear translocation of PABP4 and perhaps by increase in the cytoplasmic abundance of PABP5, but these were not sufficient to prevent apoptosis of cells. Thus PABPN1 may have a novel anti apoptotic role in mammalian cells.

## Materials and Methods

### Plasmid and SiRNA

The Stealth RNAi sequence used for silencing PABPN1 expression was the same as described earlier [16]; the control siRNA sequence (AUUACUUGGCAAUCAAAUAAGGCCC) was designed from the 5′UTR region of PABPN1 sequence (NM_004643.3), and si RNAs were purchased from Invitrogen. The reporter plasmid used for co-transfection was pCMV-SPORT-β-Gal (Invitrogen, Life Technology).

### Cell Culture and Transfection

HeLa and HEK293 cells were grown in Dulbecco’s Modified Eagle’s medium (DMEM) containing 1% glutamine (Lonza, Walkerville, MD USA) and 10% FBS (VWR, Mississauga, ON, Canada) at 37°C in presence of 5% CO_2_. Approximately 30% confluent cells, grown on 35 mm dishes were transfected with double stranded si RNA using lipofectamine2000™ (Invitrogen, Burlington, ON, Canada) according to manufacturer’s instructions. In short, for each 35 mm plate 1 µl of siRNA (20 µM annealed duplex) was mixed with 5 µl lipofectamine2000™ and 500 µl of Opti-MEM (Invitrogen, Burlington, ON, Canada) medium and incubated for 20 min before being added to the culture dish. After 24 h, the medium containing the RNA-lipofectamine complex was replaced by fresh DMEM containing 10% FBS and 1% glutamine. For co-transfection of the cells, 1 µg of plasmid was mixed with siRNA, and transfection was performed as described above. Cells were harvested between 45–80 h after transfection depending on the experiment.

### Preparation of Nuclear Extract

For isolation of nuclear proteins, cells grown on a 35 mm plate were washed three times with PBS (137 mM NaCl, 2.7 mM KCl, 4.3 mM Na_2_HPO_4_, 2H_2_O, 1.47 mM KH_2_PO_4_, pH 7.4). 200 µl of cytoplasmic lysis buffer (100 mM Tris-HCl, 100 mM NaCl, 2.5 mM MgCl_2_, 0.5% TritonX-100, pH 7.6) was added to the plate and incubated for 10–15 min on ice. Cells were scrapped off and the cell suspension was passed through a 30 gauge needle until 90% cells were lysed as judged by the presence of cytoplasmic tag free nuclei. Cell lysates were centrifuged at 2500 g for 7 min to obtain the cytoplasmic fraction. The pellet (nuclear fraction) was washed 3 times with the lysis buffer and lysed in (a) 150 µl of Laemmli buffer (50 mM Tris-HCl pH 6.8, 2% sodium dodecyl sulphate (SDS), 10% (v/v) glycerol, 5% (v/v) β-mercaptoethanol, 0.01% (w/v) bromophenol blue) for protein extraction or (b) in 1 ml of Trizol (Life technologies, Invitrogen) for RNA extraction.

### Western Blotting, Immunofluorescence Confocal Microscopy and *in situ* Hybridization

Western blotting, immunostaining, and *in situ* hybridization were performed as described previously [22], [23]. After immunostaining with PABP4 antibody, the nuclei of the cells were further stained with DAPI (Santa Cruz Biotechnology, CA, USA) (0.5 µg/µl) for 10 min, washed twice with PBS and mounted onto slides. Western lightening TM chemiluminescence reagent Plus (Perkin Elmer LAS, Shelton, USA) was used for detection of proteins on blots. The Image J Analyzer software program was used for quantitative densitometric analysis. Leica Microsystems Confocal Laser Scanning Microscope (CLSM, Microsystems, Inc., Heidelberg, Germany) equipped with a Plan-Achromat 63×/NA1.4 objective was used to visualize and image the immunostained cells.

### RNA Extraction and RT-PCR

Cells were washed three times with PBS and used for isolating total cellular RNA using the Trizol (Life Technologies, Indianapolis, IN, USA) according to manufacturer’s instructions. The quality and quantity of the RNA were determined by 1.5% agarose gel electrophoresis and spectrophotometric measurements respectively. The levels of specific mRNAs were determined by RT-PCR. An aliquot of total RNA (100–500 ng) was reverse transcribed using High Capacity cDNA transcription kit (Applied Biosystems, Life Technologies). After the reaction, 2 µL of the cDNA sample was amplified by PCR in a total master mix (Fermentas, Amherst, NY, USA) reaction volume of 50 µL, which included 100 ng of primers specific for diffeent mRNAs (Sigma, Oakville, ON, Canada) [22]. Primers used have been described previously [22], [24]. The amplification was performed using an initial denaturation step at 95°C for 4 min, and depending on the cellular level of the individual mRNA, it was followed by 30–35 cycles of denaturation at 95°C for 20 s, annealing ranging from 55–60°C, depending on the primer for 20 s and extension at 72°C for 20 s. Samples were analyzed by 1% agarose gel electrophoresis and the relative expression values of all mRNAs were normalized by the β-actin mRNA level. The scanned images of the PCR products following agarose gel electrophoresis were quantified by using image J software. For each mRNA the dose dependence of the PCR product was analyzed by using different amount of the input cDNA and 30 cycles of amplification (Fig. S1). For all the mRNAs examined in our studies 30 cycles of amplification under our experimental conditions were within the linear range. As negative controls RNA samples were subjected to PCR without the reverse transcription reaction.

### Immunoprecipitation of RNA-Protein Complex and Nuclear Proteins

Immunoprecipitation was done as previously described [23] with the following modifications. Following 72 h of transfection, cells grown in 60 mm plate were lysed in 400 µl of chilled PLC buffer (10% glycerol, 50 mM Hepes–HCl, pH 7.5, 150 mM NaCl, 1.5 mM MgCl_2_, 1 mM EGTA, 100 mM NaPPi, 100 mM NaF, and 1% Triton X-100) by gentle sonication using XL2020 Sonicator (Mandel Scientific) at setting 1, pulse 3 (4 s each) to break the cytoplasmic membrane. Cells were then centrifuged at 2500 g for 10 min to separate the nuclear fraction (pellet). The pellet was resuspended in PLC buffer with 1% formaldehyde (Electron Microscopy Sciences) for 10 min at room temperature (RT) to crosslink the RNA and proteins of RNP complexes. The nuclear pellet was then incubated in 0.25 M of glycine, centrifuged and washed three times with PLC buffer. The nuclear pellet was finally resuspended in 400 µl of chilled PLC buffer supplemented with protease inhibitor (Roche) and again sonicated at a higher setting (setting 3, 5 pulses 5 s each). Cell debris was removed by centrifugation at 12000 rpm for 10 min. The nuclear extract was incubated overnight with PABPC4 or PABPN1 antibody and rabbit or goat IgG-Agarose beads (Sigma). Following Immunoprecipitation, beads were washed extensively with PLC buffer and the RNA-protein cross-link was reversed by incubation at 70°C for 45 min in 100 µL of elution buffer (50 mM Tris-HCl, 5 mM EDTA, 10 mM DTT, 1% SDS). RNA was then extracted with three volumes of a mixture of phenol/chloroform (50/50). The RNA was precipitated using one volume of ethanol, and left overnight at −20°C. The precipitated RNA was collected by centrifugation at 10,000 g for 10 min at 4°C and resuspended in RNAse free water. Contaminating DNA was removed from RNA samples by RQ1 RNase-free DNAse (Promega) treatment prior to being reverse transcribed and PCR amplified using β-actin pre-mRNA primers [23]. For IP with PABPN1, 30 cycles of amplification were used, whereas for IP with PABP4 40 cycles were used. The samples were analyzed by electrophoresis in a 1% agarose gel.

For immunoprecipitation studies nuclear extracts were prepared as described above and incubated with the designated antibody and appropriate IgG agarose beads for 16 h at 4°C. The eluted samples and nuclear extracts were subjected to SDS-PAGE and Western blotting.

### PAT Assay

PAT assay used to determine the poly(A) tail length was done as described previously by Salles and Strikeland 1999 [25]. In short, 10 ng of total RNA was ligated with 20 ng of phosphorylated (dT)_12–18_ in a reaction containing 0.1 M DTT, 10 mM dNTP, 10 mM ATP, SuperscriptH^−^ buffer, and 10 U/µl T4 DNA ligase (New England Biolabs). The resultant mixture was hybridized with oligo dT anchor (5′GCGAGCTCCGCGGCCGCGT_12_ 3′) followed by reverse transcription with RNAseH^−^ reverse transcriptase (Invitrogen). The cDNA was then PCR amplified with oligo dT primer and β-actin specific primer (CAC ACA GGG GAG GTG ATA GCA).

### Measurement of Protein Synthesis

Protein synthesis was measured by radioactively labelling cellular proteins with [^35^S]-methionine. For this, the cells were pre-incubated in methionine free DMEM medium (Invitrogen, Burlington, ON, Canada) with 10% dialyzed FBS (Invitrogen, Burlington, ON, Canada) for 2 h at 37°C to reduce the pool of endogenous methionine. Samples were incubated with 50 µCi [^35^S]-Methionine (MP Biomedicals, Santa Ana, California) in 1 ml of fresh methionine free DMEM medium with 10% dialyzed FBS (Invitrogen, Burlington, ON, Canada) at 37°C for 30 min. Following incubation, cells were lysed with 200 µL of Lamelli buffer. The samples were boiled for 5 min and separated by SDS-10% polyacrylamide gel electrophoresis (PAGE). The gel was fixed in 10% trichloroacetic acid (TCA), at 95°C and allowed to cool down for 5 min at RT. The gel was then washed twice with 10% TCA at 20°C, and twice with 10% ethanol and finally with 100% ethanol. The gel was then soaked in 1 M Na-Salicylate for 30 min and dried at 50°C for one hour, and autofluorographed with an X-ray film for the desired time before being developed.

### Radioactivity Measurement

10 µL of each sample was spotted on small strips of filter paper. The filter paper was then placed in 100 mL of 10% TCA, and brought to boil and allowed to cool for 10 min; this process was then repeated three times. The filters were washed twice with 10% TCA at RT followed by 100% ethanol. The filter papers were placed in scintillation vials with sufficient amount of liquid scintillation counting fluid and counted using a liquid scintillation counter at a setting for [^35^S] isotope.

### Detection of Apoptosis

To detect normal, apoptotic and necrotic cells, acridine orange/ethidium bromide (AO/EB) double staining was used as previously described [26]. Cells were classified as normal, apoptotic and necrotic as described by Baskic et al., 2006 [27]. Apoptotic cells with fragmented nuclei were detected by staining the DNA of the cells with DAPI.

### Antibodies Used

PABP, SKIP, β-actin, GAPDH, p53, p-p53, GRP78, eIF2α, splicing factor, Hsp70/Hsc70, CPSF1, goat anti-mouse, rabbit, mouse and goat IgG-HRP, Texas-red-conjugated secondary antibodies were from Santa Cruz Biotechnology, Inc., CA, USA. PABPN1 antibody was from Abcam, MA, USA; PUMA antibody was from Cell Signaling Technology, Inc., Denver, USA; PABPC4 was purchased from Novus Biologicals, Canada, ULC; PABP3, 5 were obtained from Abnova, Taiwan. PABP5 used for Immunostaining was from Santa Cruz Biotechnology, Inc., CA, USA.

### Statistical Analysis

Data were expressed as mean ± SD, and the significance was assessed by one way ANOVA. A value of *P<0.05* was considered significant.

## Results

### PABPN1 Depletion

**Figure 1:**
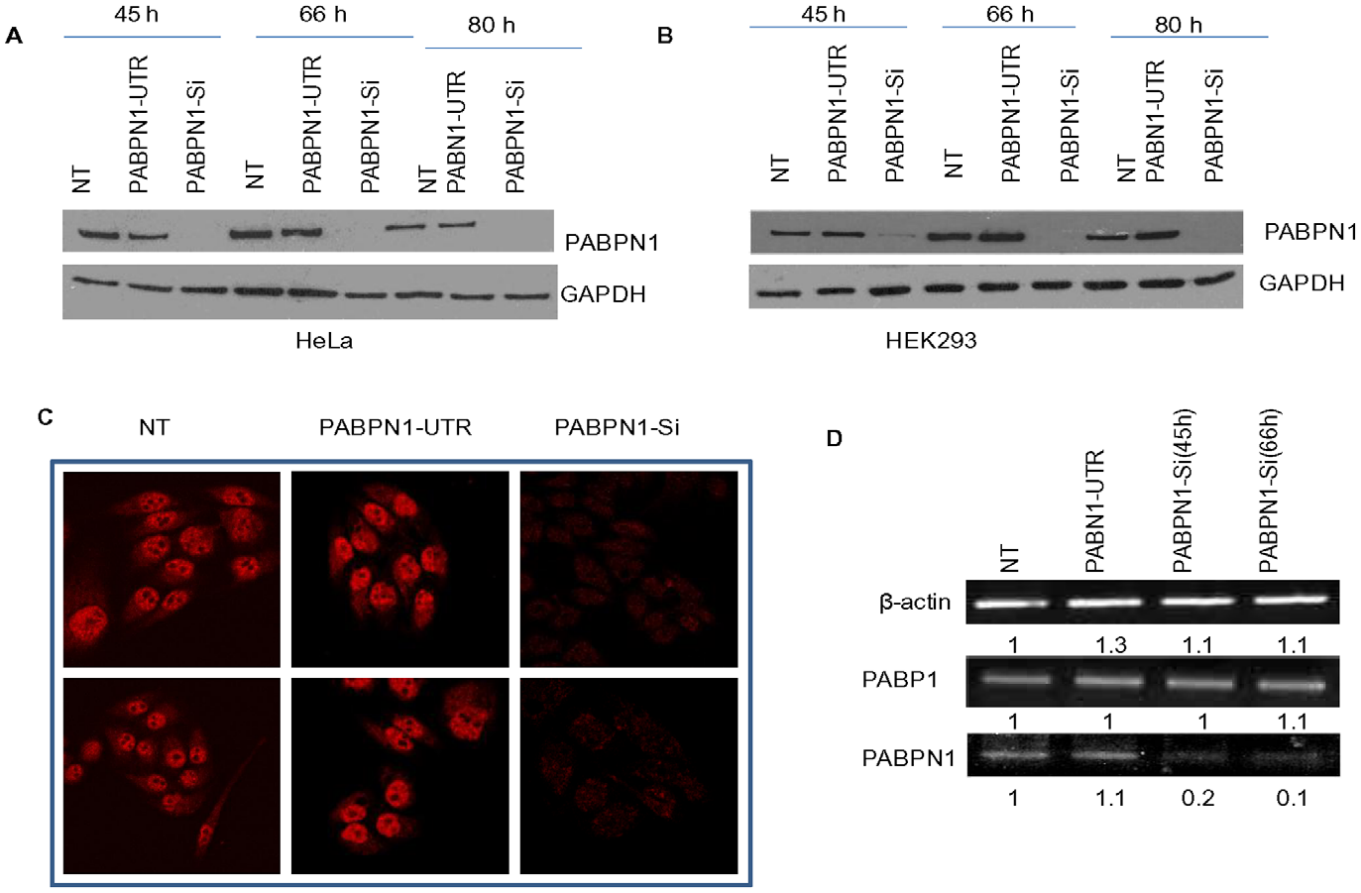
Knockdown of PABPN1 in human cells. HeLa and/or HEK293 cells were grown on 35 mm plates to approximately 30% confluence and transiently transfected with PABPN1-Si or PABPN1-UTR RNAi for indicated length of time. Non transfected (NT) control cells were also maintained in culture for the same duration as transfected cells. (A, B) Following transfection, cells were harvested at 45 h, 66 h and 80 h in Laemmli buffer. Whole cell extracts from Si, UTR, and NT HeLa (A) and HEK293 (B) cells were analyzed for PABPN1 protein by western blotting; GAPDH was used as the loading control. (C) Forty five hours after transfection, HeLa cells grown on coverslip were fixed with Para-formaldehyde and processed for immunostaining with PABPN1 specific antibody and counterstained with Texas-red-conjugated secondary antibody. Processed specimens were then examined with a confocal microscope. Images from two different sections of the slide are shown here. (D) Forty five hours and sixty six hours after transfection, RNA was extracted from HeLa cells using Trizol and reverse transcribed; cDNA thus made were used for PCR with primers specific to β-actin, PABP and PABPN1. Samples from PABPN1-UTR Si and non transfected (NT) cells were collected at 66 h time point. The bands on the gel representing the PCR products were scanned and quantified as described in Materials and Methods. The band intensities are shown in arbitrary unit.

Several biochemical studies have implicated a role of PABPN1 in assisting poly(A) polymerase in the addition of poly(A) tracts at the 3` end of mRNA, and as a ruler to control the length of the poly(A) tract to approximately 250 adenines [28]. However, little is known about PABPN1’s *in vivo* cellular functions. To address this, we used RNA interference (RNAi) to deplete PABPN1 from human cell lines. We show here (Fig. 1 A, B) that the RNAi targeted to a sequence within the coding region of PABPN1 mRNA (PABPN1-Si) produced significant reduction of PABPN1 abundance in both HeLa and HEK293 cells. On the other hand RNAi targeted to a 5′ UTR sequence (PABPN1-UTR) did not show any inhibition of PABPN1 expression. Maximum depletion of cellular PABPN1 level was observed following 45–80 hours treatment. Further immunofluorescence studies (Fig. 1C) showed that more than 90% of HeLa cells had reduced level of PABPN1 following PABPN1-Si treatment. Measurement of PABPN1 mRNA level also showed approximately 90% reduction of its abundance in PABPN1-Si treated cells, whereas no reduction of PABPN1 mRNA level was observed following the PABN1-UTR treatment. Similarly the abundance of non-targeted β-actin mRNA remained unchanged after PABPN1-Si RNA treatment (Fig. 1D). Since the RNAi targeted to the 5′ UTR produced no inhibition of PABPN1 expression we used this PABPN1-UTR as a control in subsequent experiments.

**Figure 2:**
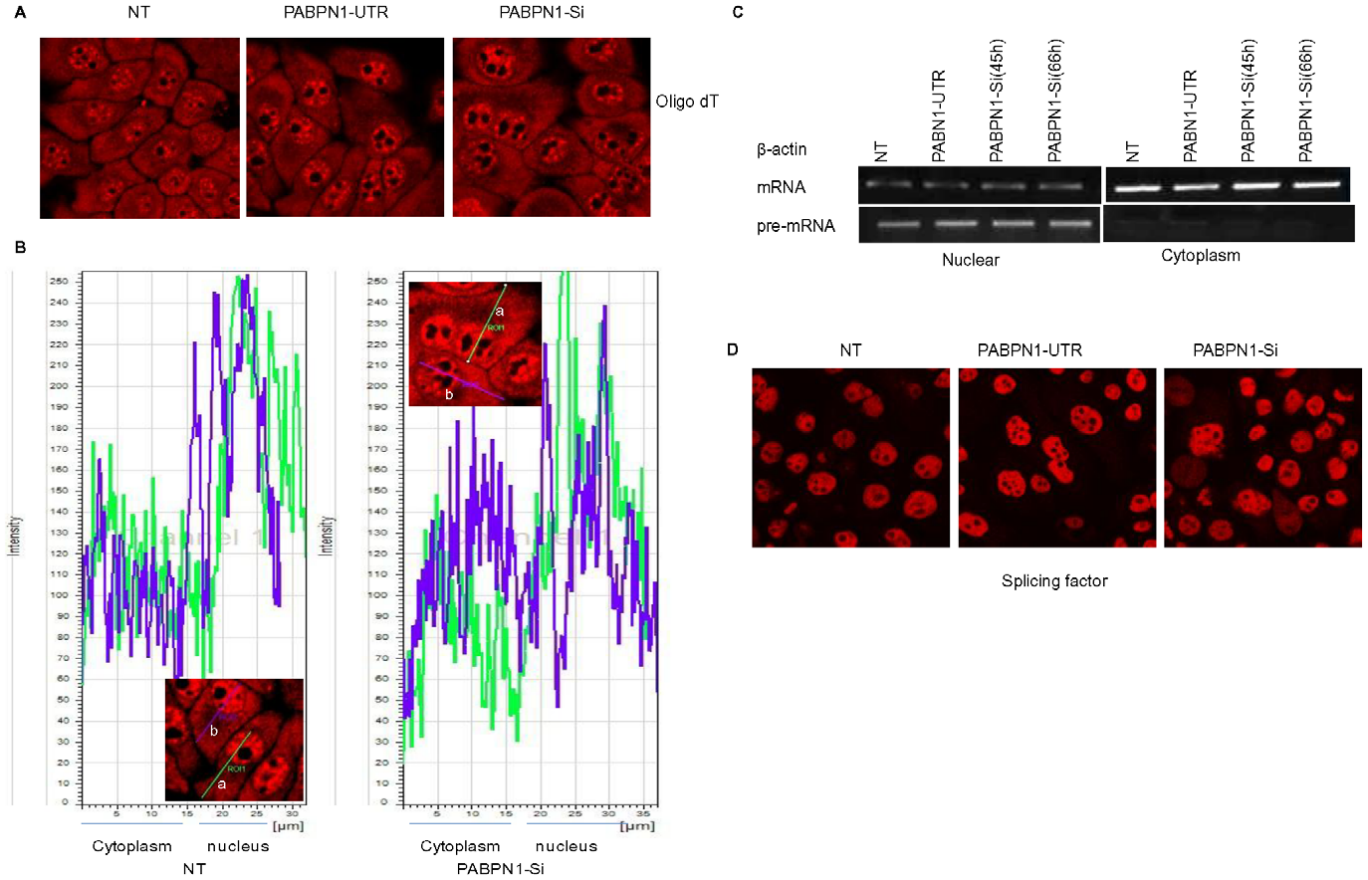
PABPN1 depletion in human cells does not affect cellular transcription and mRNA export. HeLa cells were grown on 35 mm plates and transiently transfected with PABPN1-Si or PABPN1-UTR RNAi. (A) After 45 h of transfection, cells grown on cover slips were fixed with Para-formaldehyde and hybridized with Cy3 labeled oligo-dT probes. Processed specimens were then examined with a confocal microscope. (B) The fluorescent signals in nucleus and cytoplasm of two different cells (a-green; b-purple line scans) from (A) were quantified by line scans using the Leica software. (C) RNA was extracted from the nuclear and cytoplasmic fractions of 45 and 66 h transfected and non transfected (NT) cells. Distribution of β-actin pre-mRNA and mRNA transcripts in nuclear and cytoplasmic fractions were analyzed by RT-PCR. (D) Forty five hours after transfection, HeLa cells grown on cover slips were fixed with Para-formaldehyde and processed for immunostaining with splicing factor specific antibody and counterstained with Texas-red-conjugated secondary antibody. Processed specimens were then examined with a confocal microscope.

As PABPN1 is considered to be important for mRNA export from the nucleus, we further examined whether depletion of PABPN1 leads to nuclear accumulation of mRNA. Presence of mRNA in intact cells was detected by immuno fluorescence confocal microscopy using a fluorescently labeled oligo (dT) probe [22]. Results show that there was no detectable increase of nuclear fluorescence in PABPN1 depleted cells (Fig. 2A). To obtain a quantitative estimate of nuclear and cytoplasmic distribution of poly(A) containing mRNAs, line scans of the images were performed. The relative distribution of mRNAs between the nuclear and cytoplasmic fractions was not affected by depletion of PABPN1 (Fig. 2B). Further measurement of the cytoplasmic abundance of β-actin mRNA by RT-PCR confirmed that the cytoplasmic level of this mRNA was not affected by depletion of PABPN1 (Fig. 2C, top row). Thus the results of both global and a specific mRNA distribution (Fig. 2, panels A–C) suggest that PABPN1 depletion did not affect accumulation of mRNA in the cytoplasm.

The potential effect of PABPN1 depletion on the poly adenylation of mRNA could also affect global transcription; hence, we examined transcription of an essential mRNA (β-actin) in HeLa cells. This was performed by measuring the abundance of pre-mRNA transcripts using intron specific primers in RT- PCR assay (Fig. 2C, bottom row). Results show that non-transfected (NT), PABPN1-UTR and Si-PABPN1 transfected cells have similar levels of pre-mRNA for β-actin. Additional immunofluorescence studies using an antibody to splicing factor 1 also showed (Fig. 2D) that its distribution in nuclear speckles was not influenced by PABPN1 depletion, suggesting a lack of effect of PABPN1 depletion on cellular transcription and splicing.

**Figure 3:**
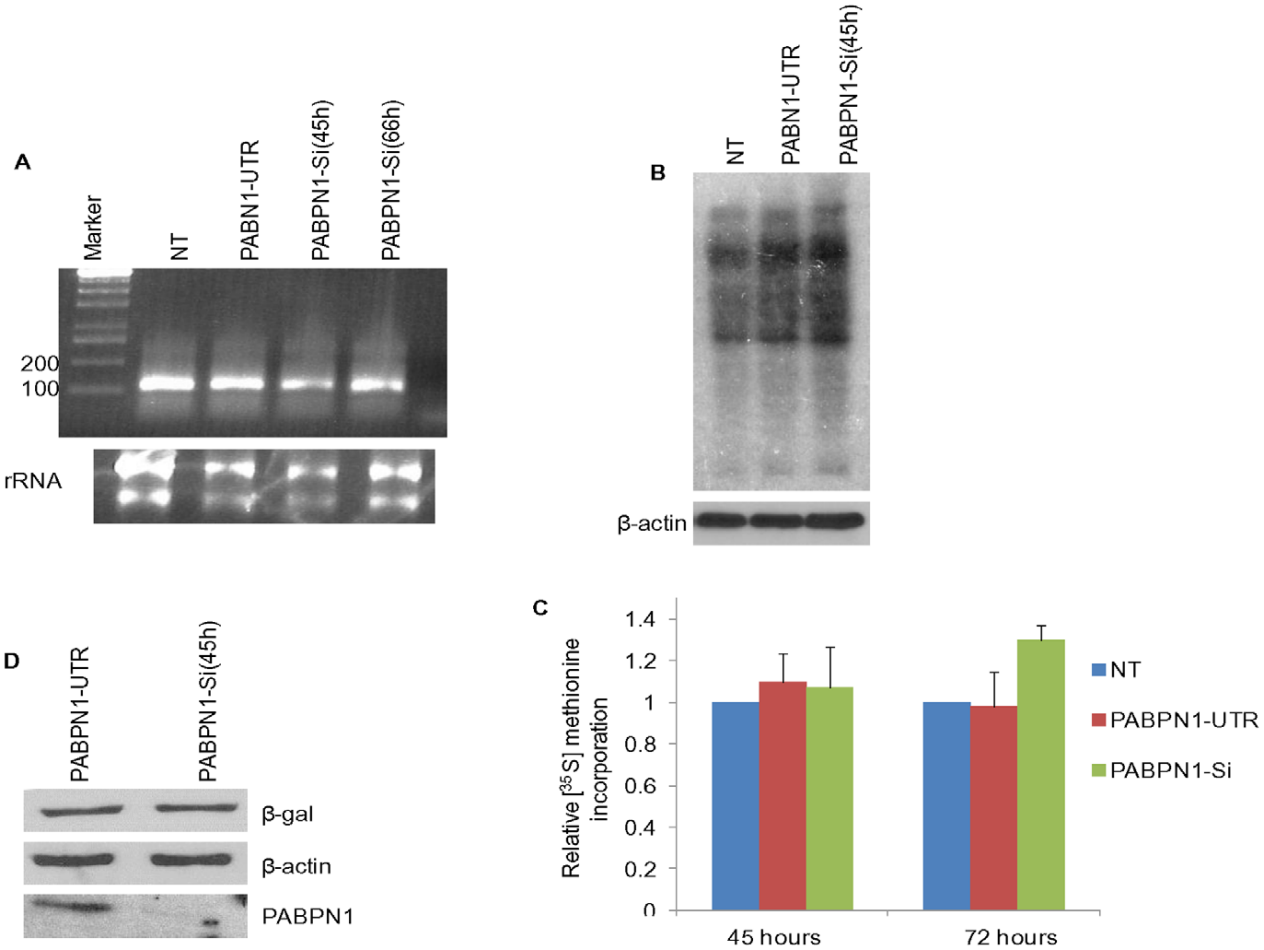
PABPN1 depletion in HeLa cells does not affect β-actin poly(A) tail length and cellular translation. HeLa cells were grown on 35 mm plates and transiently transfected with PABPN1-Si or PABPN1-UTR RNAi. (A) Total RNA was extracted following 66 h of transfection with SiRNA and from non transfected (NT) cells and poly(A) tail length was analyzed by PAT assay as described by Salles et al. 1999 [23] with β-actin specific primers. The PCR product was analyzed in 1% agarose gel. (B) Forty five hours after transfection, live cells were labeled with [^35^S]-methionine, total protein was extracted and subjected to 10% PAGE and autoflurographed. Samples were also used for measuring β- actin abundance by western blotting as loading controls. (C). The same samples along with 72 h samples were used for measuring [^35^S] - methionine incorporation as described in Materials and Methods. Results are shown as incorporations relative to what was seen for non transfected cells after normalizing for the difference in cell numbers by β-actin levels. The average of three separate experiments is shown. (D) Hela cells were cotransfected with RNAi and CMV-β-gal plasmid. Level of β-galactosidase was assessed by western blotting; β-actin and PABPN1 levels were used as loading and transfection controls respectively.

We also examined whether PABPN1 depletion would affect the length of the poly(A) tract of mRNA by measuring it for β-actin mRNA using an anchored oligo (dT) and mRNA specific primers in RT-PCR based PAT assay [25]. The result show that the ability to add poly(A) was not affected by PABPN1 depletion since the average length of the poly(A) tract of β-actin mRNA was approximately 125–150 nucleotides in both PABPN1-Si and PABPN1-UTR cells (Fig. 3A).

Whether or not depletion of cellular PABPN1 could affect global protein synthesis was examined by comparing incorporation of [^35^S]-Methionine into polypeptides. Results of analyses of protein synthesis by SDS-PAGE followed by auto fluorography of equivalent amount proteins from NT, UTR and Si-PABPN1 transfected cells showed that there was no detectable difference in protein synthesis amongst these samples (Fig. 3B). Results of total incorporation of [^35^S]-Methionine into TCA insoluble protein precipitates also showed no decrease in total protein synthesis in PABPN1 depleted cells (Fig. 3C). However, a small but reproducible increase in global protein synthesis was observed in these cells. In addition to global protein synthesis we also compared the expression of β-galactosidase reporter gene by co-transfecting PABPN1-Si treated cells with a CMV-SPORT-β-gal plasmid. Results showed (Fig. 3D) no significant difference in our samples. The results of reporter gene expression also support our results of specific mRNA abundance and intron specific primer directed RT-PCR (Fig. 2C) studies that PABPN1 depletion had no detectable effect on mRNA transcription, processing and transport in HeLa cells. Studies with HEK 293 cells also produced similar results.

### Compensatory Effect of Cytoplasmic PABP Family Members

**Figure 4:**
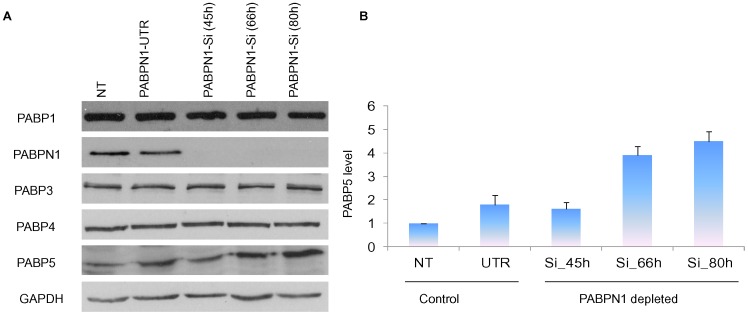
Level of different cellular PABPs in PABPN1 depleted cells. HeLa cells were grown on 35 mm plates and transiently transfected with PABPN1 Si-RNA or PABPN1-UTR RNAi (A) Following transfection, cells were harvested in Laemmli buffer at 45 h, 66 h and 80 h. Whole cells extracts from NT (66 h), UTR (66 h), and PABPN1 Si (45 h, 66 h, and 80 h) transfected cells were analyzed for PABP1, 3, 4, 5 and PABPN1 by western blotting. GAPDH was used as loading control. (B) PABP5 abundance was calculated by scanning band intensities and expressed as an arbitrary unit. Data are mean ± SE, P<0.05, n = 3.

Considering that several years of extensive investigations using *in vitro* approaches have suggested a role of PABPN1 in the poly adenylation process it was surprising that PABPN1 depletion in cultured human cell lines had no detectable effect on mRNA abundance and translation in two human cell lines. Since, other cytoplasmic PABP family members could have redundant cellular functions we examined the abundance of several PABP family members by western blotting. Results of our studies showed that PABPN1 depletion had no effect on the abundance of PABP1, PABP3 and PABP4 (Fig. 4A). However, the cellular level of PABP5 was 4–5 fold higher in PABPN1-Si treated cells than what was observed in NT cells (Fig. 4A, B). There was also some increase of PABP5 level in PABPN1-UTR treated cells which could be due to a weak effect of PABPN1-UTR on PABPN1 level. Further control studies using mock transfected and another random Si (Si control) showed no effect on PABP5 abundance (result not shown).

**Figure 5:**
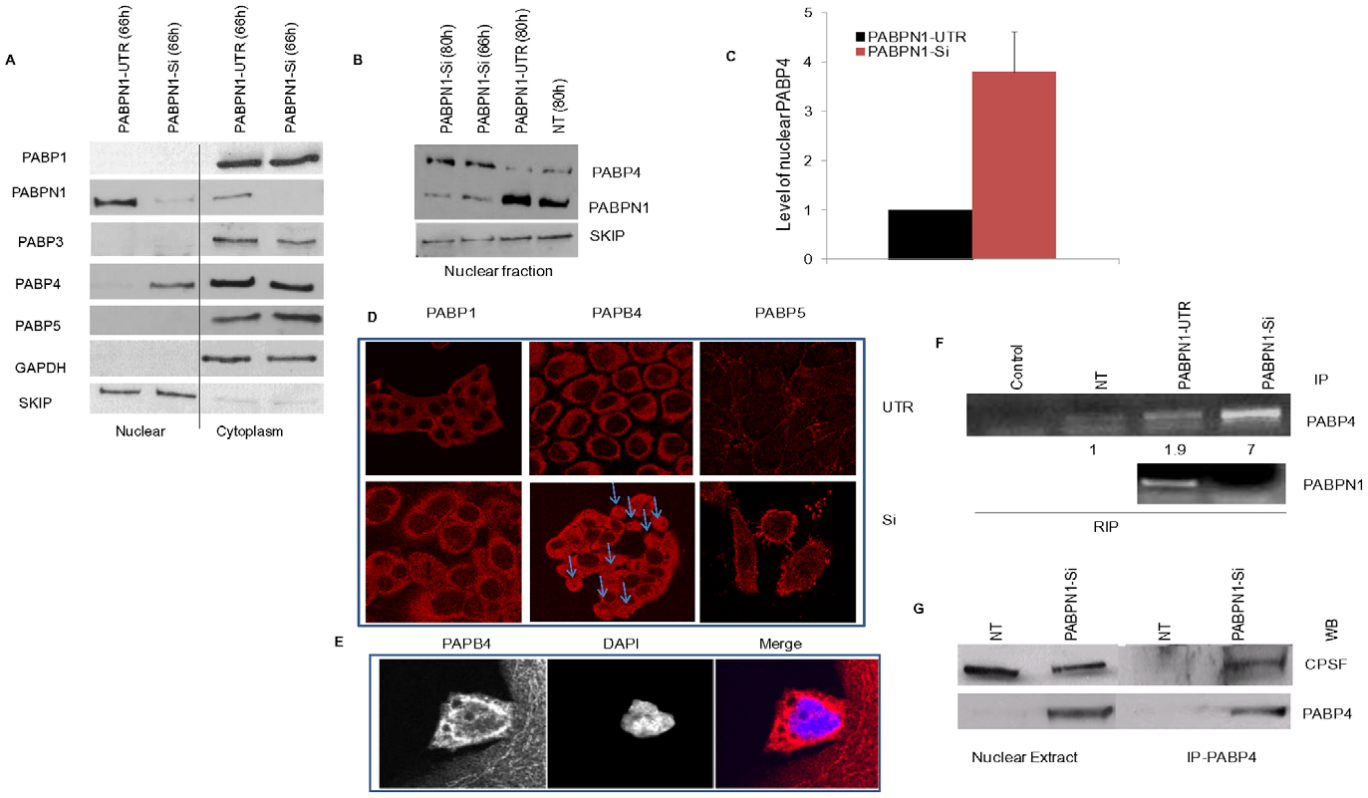
Cytoplasmic-nuclear distribution of different cellular PABPs. HeLa cells were grown on 35 mm plates and transiently transfected with PABPN1 Si-RNA or PABPN-UTR RNAi (A) Sixty six hours after transfection, cytoplasmic and nuclear extracts were prepared from UTR, and Si-PABPN1 cells and probed with PABP1, 3, 4, 5, and PABPN1 antibodies. GAPDH and SKIP (loading control) were used to determine cross contamination in each fractions. (B) Nuclear fractions from non transfected (NT), PABPN1-Si and UTR-Si transfected cells were used for western blotting with PABP4, PABPN1, and SKIP (loading control) antibodies as previously described. (C) Nuclear PABP4 abundance was calculated by scanning band intensities and expressed as an arbitrary unit. Data are mean ± SE, P<0.05, n = 3. (D) Following 66 h of transfection with Si RNAs HeLa cells grown on cover slips were fixed with Para-formaldehyde and processed for immunostaining with PABP1, 4, 5 specific antibodies and counterstained with Texas-red-conjugated secondary antibody. (E) The PABP4 stained slide was further stained with DNA staining dye, DAPI, to show the nuclear localization of PABP4 in PABPN1-Si transfected cells. Processed specimens were then examined with a confocal microscope (F) The nuclear fractions of NT, PABPN1-UTR and PABPN1-Si transfected cells after 66 h of treatment were immunoprecipitated with PABP4 antibody; the RNA bound to PABP4 was extracted, and analyzed by RT-PCR with β-actin pre-mRNA specific primers as described in legends to figure 1. The band intensities are shown in arbitrary unit. Immunoprecipitation with PABPN1 antibody was used as a positive control. The control lane indicate PCR product of immunoprecipitated RNA from PABPN1-Si cells without reverse transcription. The lanes at the bottom panel show β-actin pre-mRNA immunoprecipitated with PABPN1 antibody from PABPN1-UTR and PABPN1-Si transfected cells as controls. (G) Immunoprecipitation from the nuclear extract was carried out with PABP4 antibody as described in Materials and Methods. The agarose bead eluted (IP-PABP4) and cell equivalent amount of the input nuclear extract were used for western blotting (WB) with CPSF and PABP4 antibody.

To test whether cytoplasmic PABPs could translocate to the nucleus and perhaps compensate for the loss of PABPN1, we examined the cytoplasmic and nuclear distribution of different cytoplasmic PABPs in PABPN1-UTR and PABPN1-Si transfected cells. Results show that PABP1, PABP3 and PABP5 were present almost entirely in the cytoplasmic fractions of both PABPN1-UTR and PABPN1-Si treated cells (Fig. 5A). A small decrease in cytoplasmic distribution of PABP3 seen in PABPN1-Si treated cells (Fig. 5A) could be due to difference in loading as judged by the GAPDH level. In contrast, a low level of PABP4 was detectable in the nuclear fractions of both NT and PABPN1-UTR transfected cells (Fig. 5A & B). However, approximately four folds increase of PABP4 distribution in the nuclear fraction of PABPN1 depleted cells was observed (Fig. 5 A, B & C). To test for cross contamination between nuclear and cytoplasmic fractions we examined the sub cellular distribution of two nuclear and cytoplasmic marker proteins SKIP and GAPDH respectively. Results (Fig. 5A) show that there was no appreciable cross contamination of our sub cellular fractions.

Nuclear localization of PABPs was also examined by immunofluorescence confocal microscopy.

It showed that while PABP1 and PABP5 were almost exclusively localized in the cytoplasm of PABPN1-UTR transfected cells, a significant proportion of PABP4 localized to the nucleus of PABPN1 depleted cells (Fig. 5D). However, no detectable level of PABP1 or PABP5 was present in the nucleus of the same (Fig. 5D). Nuclear localization of PABP4 was further confirmed by co-immunofluorescence studies using DAPI to stain the cell nucleus (Fig. 5E). We then examined whether the nuclear PABP4 was associated with the poly(A) tract of mRNAs. This was performed by using a modified RNA CHIP assay. In short, after sub cellular fractionation the nuclear were subjected to immunoprecipitation with PABPN1 or PABP4 antibody. Analysis of the immunoprecipitated RNA samples by RT PCR using β-actin intron specific primers showed the presence of β-actin pre-mRNA transcript in the immunoprecipitates of both controls (NT & PABPN1-UTR) and PABPN1-Si treated cells. However, approximately four fold more β-actin pre-mRNA transcript was immunoprecipitated from PABPN1-Si treated cell extract than what was immunoprecipitated from PABPN1-UTR treated cell extract (Fig. 5F). Thus the level of increased binding of PABP4 to β-actin pre-mRNA corresponds to the increase of PABP4 localization in the nucleus of PABPN1-Si treated cells (Fig. 5, A–C).

Since PABPN1 is known to interact with CPSF during poly(A) tract addition (8) we tested whether the nuclear PABP4 in PABPN1 depleted cells also interacts with CPSF. The results of co-immunoprecipitation studies using PABP4 antibody show that CPSF1 was immunoprecipitated by PABP4 antibody from the nuclear extract of PABPN1-Si treated cells but not from the same of NT cells (Fig. 5 G). As positive control we tested for immunoprecipitation of PABP4 by its own antibody. Results show that PABP4 was present in the immunoprecipitated samples. Judging from the input levels of CPSF it was evident that a significant level of CPSF was associated with PABP4 in PABPN1 depleted cells (Fig. 5 G).

### Apoptosis of PABPN1 Depleted Cells

**Figure 6:**
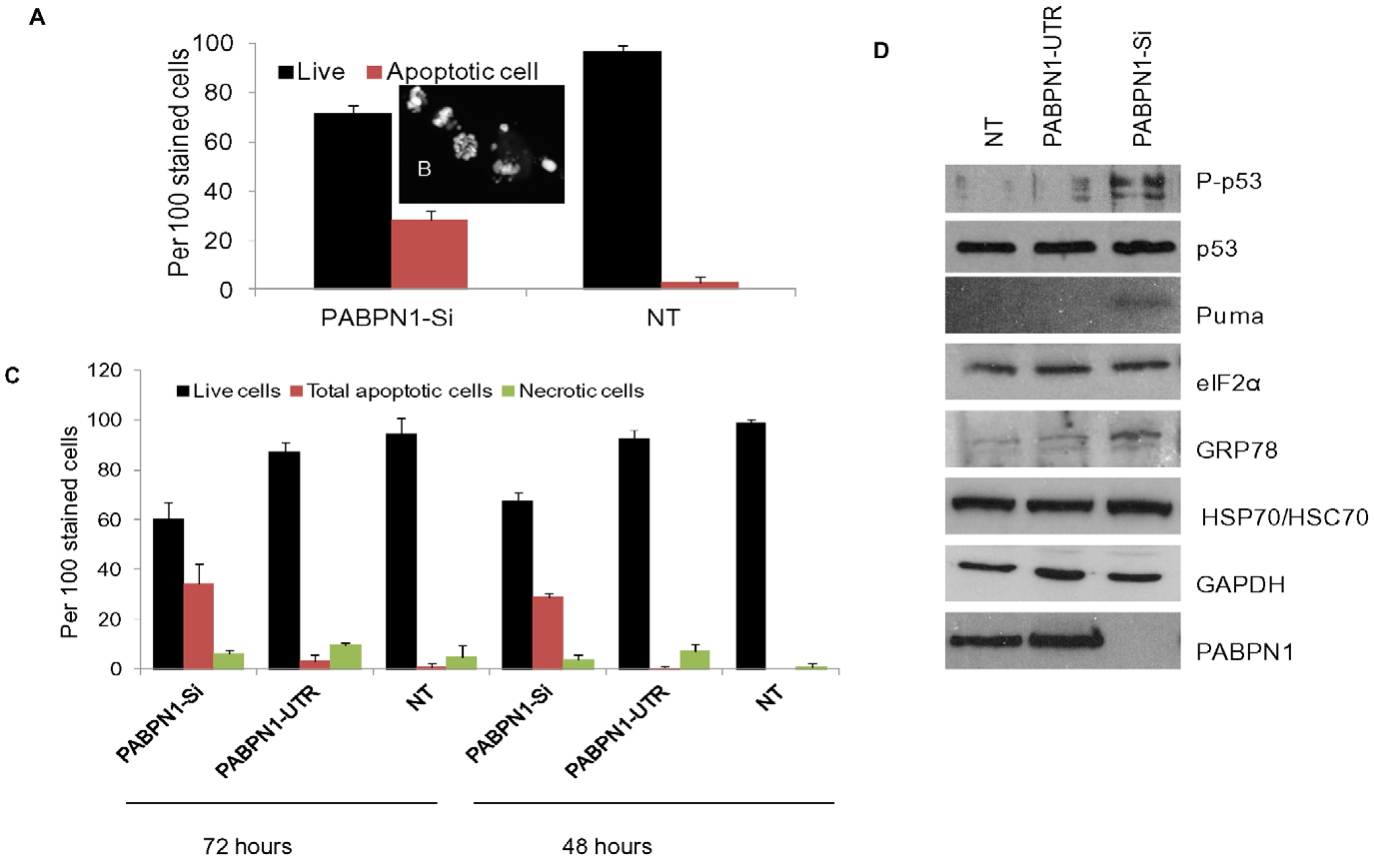
Cell death in PABPN1 depleted Hela cells. (A) After 72 h of transfection, cells on coverslip were stained with DAPI and observed under the microscope; cells which had fragmented/condensed nuclei were counted as apoptotic cells. (B) Confocal image of DAPI stained cells showing fragmented/condensed nuclei. (C) After 48 and 72 h of transfection, HeLa cells were trypsinized, stained with EB/AO and scored for the percentage of live, apoptotic and necrotic cells. Approximately 100 cells were examined and scored. Results are mean ± SE, P<0.05, n = 2. (D) Whole cell extracts from 66 h PABPN1-Si, PABPN1-UTR transfected and non transfected (NT) were analyzed by western blotting for different polypeptide markers for apoptosis and ER stress.

Examination of the presence of apoptotic cells by AO/EtBr and DAPI staining revealed that significantly more cells were apoptotic in the cultures following PABPN1-Si treatment than what was observed for the NT and PABPN1-UTR treated cells. After 72 hours almost 40% of PABPN1 depleted cells were undergoing apoptosis while negligible amount of cells in our control cultures were apoptotic (Fig. 6A, B). In addition, approximately 10% of transfected cells were necrotic, which probably was due to the effect of the transfection reagents. To further analyze the mechanism of apoptosis in PABPN1 depleted cells we examined the abundance of a number of apoptotic marker proteins by western blotting. As shown in Fig. 6C, there was a significant increase in the abundance of phosphorylated p53 while the abundance of total p53 remained unchanged in PABPN1-Si treated cells. The p53 target gene PUMA in PABPN1 depleted cells also showed an increase in abundance over what was observed in NT and PABPN-UTR transfected cells. PUMA is a well known apoptosis inducing protein which can cause permeabilization of mitochondria leading to cell death. We also investigated the changes in the expression of a well known marker of ER stress by western blotting. Results showed that GRP78 level increased in PABPN1 depleted cells (Fig. 6C).

Furthermore, we measured the abundance of additional markers of cell stress, such as, the constitutive HSC70 and the inducible HSP70 by western blotting with an antibody that recognizes both proteins. The result show that HSC/HSP70 abundance did not increase following PABPN1 depletion. Therefore, PABPN1 depletion produced a distinct ER stress response. In our studies the abundance of eIF2-α and GAPDH remained unchanged by PABPN1 depletion suggesting that the increase of phospho p53, Puma and GRP78 was not due to non specific effect of PABPN1 depletion on protein abundance. Taken collectively results of our studies suggest that in spite of the effort of PABP4 and PABP5 to rescue cells from the adverse effect of PABPN1 depletion, it was not sufficient to prevent cell death. Perhaps PABPN1 plays a more critical anti apoptotic function than its previously believed role in poly(A) tail elongation.

## Discussion

We tested two different target sequence of PABPN1 mRNA for SiRNA mediated ablation of PABPN1 expression. The first target was within the coding region of mRNA and the second target sequence was in the 5′UTR. Our studies demonstrated that the coding region was an effective target and the 5′UTR was probably not accessible for base pairing with the SiRNA due to presence of extensive secondary structures, therefore, did not produce any effect on PABPN1 expression. Examination of various parameters of cellular mRNA metabolism demonstrated that there were no detectable effects of PABPN1 depletion on mRNA export, mRNA abundance, and mRNA translation. In addition, cellular transcription as judged by the presence of pre-mRNA transcript of β-actin and localization of splicing factor1 in nuclear speckles was unaffected by PABPN1 depletion. Although it is well accepted that the central role of PABPN1 is to stimulate addition of poly(A) tract we did not observe any shortening of the length of the poly(A) tract. We examined the poly(A) tract length for a specific mRNA such as the β-actin mRNA, and found that the average poly(A) tract length was approximately 150–200 nucleotides for both control and PABPN1 depleted cells.

We have demonstrated that amongst the cytoplasmic PABPs only PABP5 showed a significant increase in abundance in PABPN1 depleted cells. The cellular level of other cytoplasmic PABPs including PABP3 and PABP4 remained unchanged. The significance of the increase of PABP5 abundance is not clear at this time. Since PABP5 is also capable of interacting with eIF4G albeit with a lower efficiency than PABP1 (results not shown), perhaps the increase in PABP5 abundance stimulates protein synthesis in PABPN1 depleted cells as cellular homeostasis. Interestingly, a small but reproducible increase of global protein synthesis was observed following PABPN1 depletion. Beside its ability to bind poly(A), little is known regarding the cellular function of PABP5. It is possible that PABP5 is involved in regulating the metabolism of a specific sub-family of mRNAs (18). As such, it will be important to examine whether translation and/or stability of mRNAs encoding regulators of apoptosis can be modulated by PABP5.

Although the abundance of PABP4 did not change in PABPN1 depleted cells, an increase in its nuclear localization was evident following depletion of cellular PABPN1. It was previously shown that UV damage results in nuclear translocation of both PABP1 and PABP4 [29]. However, PABPN1 depletion did not lead to nuclear accumulation of PABP1. Nuclear translocation of PABP1 and PABP4 was linked to nuclear retention of poly(A) containing mRNAs [29] but in our studies export of poly(A) containing mRNAs was not affected by PABPN1 depletion. We also demonstrated here that the nuclear PABP4 was associated with nuclear pre-mRNA transcript of β-actin. In view of the lack of any detectable effect of PABPN1 depletion on mRNA biogenesis, it is conceivable that PABP4 compensates the function of PABPN1 following its depletion. PABP4 is capable of binding to poly(A) with similar affinity as the PABP1 [30], it is therefore, not surprising that in absence of PABPN1, PABP4 binds to nuclear poly(A) tract. Furthermore, presence of PABP4 in β-actin pre-mRNA and its association with CPSF1 in PABPN1 depleted cells strongly argues in favor of a role of PABP4 in the poly adenylation of primary transcripts. Further studies will be necessary to determine whether PABP4 can stimulate poly(A) polymerase mediated poly adenylation of mRNA. Since in our studies we did not observe nuclear translocation of PABP1 it is likely that despite their similarities PABP4 and PABP1 responds to different cellular signals.

In spite of the cellular homeostasis by increasing PABP5 abundance and nuclear translocation of a related poly(A) binding protein almost 40% PABPN1 depleted cells were apoptotic within 72 hours. Recent evidences show that beside poly(A) addition PABPN1 has other vital functions in RNA metabolism (20, 21), such as, processing of snoRNAs (20) and selection of cleavage and polyadenylation site (21). It was shown recently that depletion of PABPN1 in mammalian cells leads to a switch to the use of the proximal polyadenylation site for a large number of cellular mRNAs resulting in transcripts with shorter 3′ untranslated region (UTR). Shortening of the 3′ UTR results in the loss of micro RNA target sites, therefore, PABPN1 is important for 3′UTR mediated repression of gene expression (21). It is likely that PABP4 only compensates some but not all the cellular functions of PABPN1.

We showed here that in response to PABPN1 depletion p53 was phosphorylated in serine 46 which is known to activate p53 for inducing expression of PUMA. We demonstrated PUMA expression is activated in PABPN1 depleted cells. Phosphorylation alters the structural conformation of p53 leading to its stabilization and activation [31]. Phosphorylation of p53 at serine 46 has been correlated with the activation of the transcriptional program of p53 culminating in cell apoptosis [32]. It has been previously shown that PUMA binds to Bcl2 relieving p53 from the p53/Bcl2 complex [33] thus allowing free p53 to bind monomeric Bax in the cytosol, causing the latter to oligomerize and induce mitochondrial outer membrane permeabilization (MOMP) [34]. The transcription dependent nuclear action of p53 cooperates with its transcription-independent, cytosolic/mitochondrial action through transactivation of the *PUMA* gene [35]. Recent studies have suggested a role of PABPN1 in regulating apoptosis [36]. Over expression of wild type PABPN1 was shown to improve survival of cells that accumulate the poly alanine expanded mutant PABPN1 [36]. PABPN1 functioned as an early anti apoptotic signal by regulating expression of X-linked inhibitor of apoptosis (XIAP). Although, depletion of PABPN1 led to down regulation of XIAP expression in COS-7 cells [36], cell viability was not affected significantly. XIAP is a multifunctional ubiquitin ligase, but how XIAP functions as a pro-survival protein is not clear. Since we did not examine XIAP expression it is not known whether XIAP is involved in apoptosis of PABPN1 depleted HeLa or HEK-293 cells. We have also demonstrated induction of glucose-regulated protein 78 (GRP78) in PABP depleted cells. GRP78 is a known marker of ER stress. GRP78 is an endoplasmic reticulum chaperone whose main function is in the process of proper folding of nascent glycoproteins [37]. GRP78 is also present on the cell surface and functions as a signaling receptor and can act as a pro survival protein. Taken together our results and those previously reported in the literature, loss of PABPN1 perhaps trigger a complex cross talk between different pro apoptotic and anti apoptotic proteins. The apoptotic signal eventually over rides other compensatory signals, including but not limiting to nuclear translocation of PABP4 and up regulation of PABP5 expression. Further detailed analyses of the mechanism of apoptosis and contributions of different cell death pathways in PABPN1 depleted cells will be carried out in the future. It will also be valuable to examine to what extent PABP4 and PABP5 compensates cellular mRNA metabolism following PABPN1 depletion by performing double knock down experiments.

## Supporting Information

Figure S1 **Dose response of PCR reaction for different mRNAs.** 250 ng of total RNA from non-transfected (NT) Hela cells was reverse transcribed as described in materials and methods, and 1, 2 and 3 µl of the cDNA was amplified using the following primers (a) β-actin (b) pre-mRNA β-actin (c) PABPN1 (d) PABP1 for 30 cycles. The scanned images of the PCR products following agarose gel electrophoresis were quantified by using image J software. The band intensities in arbitrary unit were plotted against the volume of cDNA used, and error bars were calculated from two repeats.
(TIF)
