# Supplementary figures and images for: Depletion of Nuclear Poly(A) Binding Protein PABPN1 Produces a Compensatory Response by Cytoplasmic PABP4 and PABP5 in Cultured Human Cells
Source: PLoS One. 2012 Dec 31;7(12):e53036. doi: 10.1371/journal.pone.0053036 (PMC3534090; doi:10.1371/journal.pone.0053036)

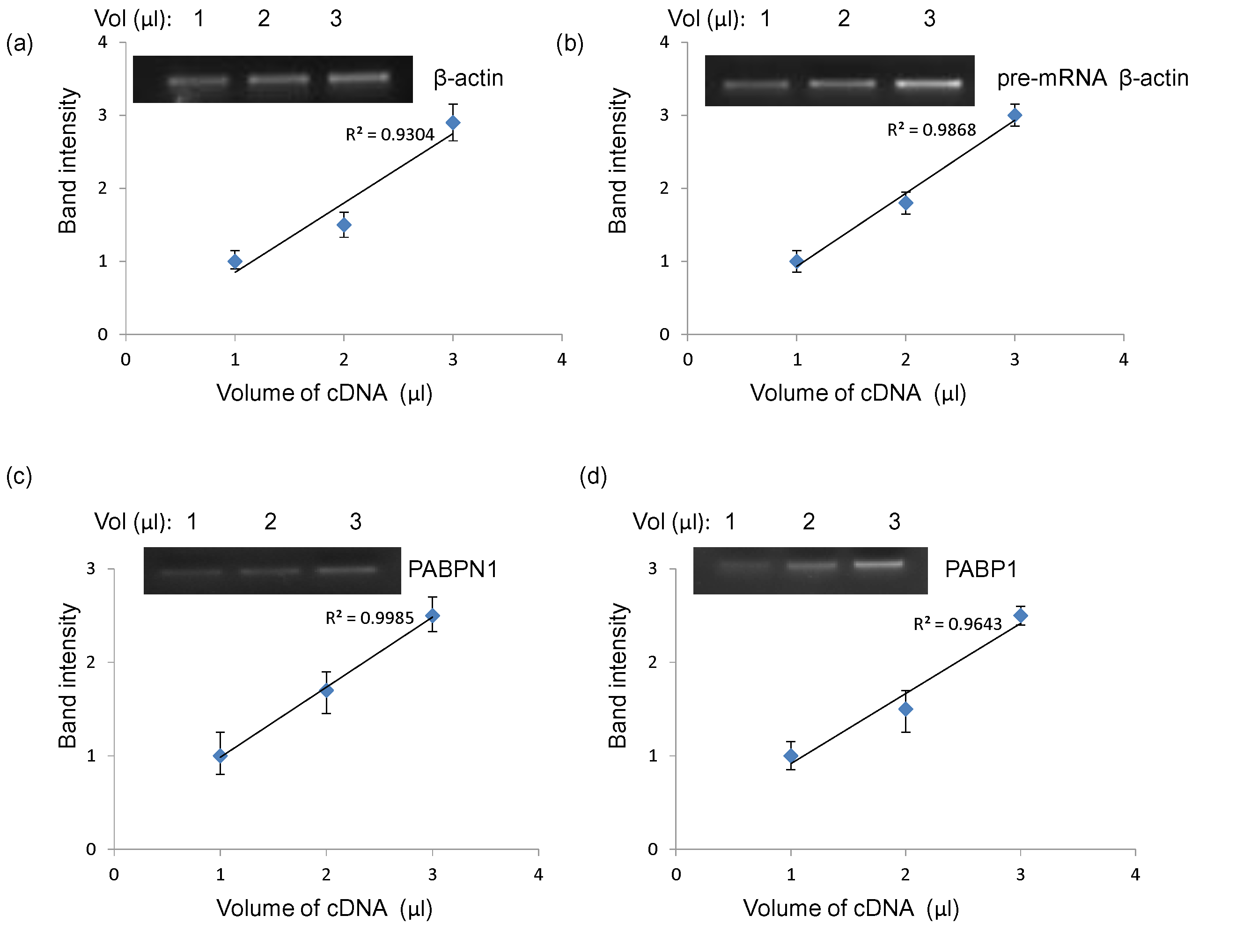

Supplement: Figure S1 — Dose response of PCR reaction for different mRNAs. 250 ng of total RNA from non-transfected (NT) Hela cells was reverse transcribed as described in materials and methods, and 1, 2 and 3 µl of the cDNA was amplified using the following primers (a) β-actin (b) pre-mRNA β-actin (c) PABPN1 (d) PABP1 for 30 cycles. The scanned images of the PCR products following agarose gel electrophoresis were quantified by using image J software. The band intensities in arbitrary unit were plotted against the volume of cDNA used, and error bars were calculated from two repeats. (TIF) [file pone.0053036.s001.tif]
